# Significance of Early Detection and Management of Trauma-Induced Inflammatory Root Resorption: A Case Report

**DOI:** 10.7759/cureus.93663

**Published:** 2025-10-01

**Authors:** Abdulaziz Altahtam, Yousra Alkhairallah, Alwaleed S Alsudais, Abdullah M Albaiz

**Affiliations:** 1 Endodontics, Al Yamamah Hospital, Ministry of Health, Riyadh, SAU; 2 Endodontics, Prince Sultan Military Medical City, Riyadh, SAU; 3 College of Dentistry, King Saud University, Riyadh, SAU

**Keywords:** dental trauma, endodontics, extrusive luxation, inflammatory root resorption, open apex

## Abstract

This case report highlights the importance of early detection and timely management of trauma-induced inflammatory root resorption (external inflammatory root resorption, EIR) following dental injuries. A nine-year-old boy presented with an extrusive luxation injury of teeth #11 and #21, accompanied by an uncomplicated crown fracture. Our proposed treatment plan was to opt for root canal treatment for both central incisors to prevent EIR; however, the patient’s guardian opted for root canal treatment only for tooth #21 and to keep tooth #11 under observation, as it was vital at the time. Unfortunately, at the eighth-week follow-up visit, signs of root resorption on tooth #11 became evident. Treatment using calcium hydroxide intracanal dressings every two weeks was initiated, successfully arresting the resorption. The root canal space was subsequently obturated with bioceramic putty. At the three-month follow-up, tooth #11 demonstrated stability of the resorption site, periapical healing, and the absence of clinical symptoms and mobility. This case highlights the crucial role of immediate intervention to preserve dental tooth structure and prevent or stop root resorption. It emphasizes that inadequate or delayed management can significantly worsen the prognosis of trauma-induced root resorption.

## Introduction

Dental trauma is defined as damage to the dentoalveolar complex. These injuries are a common occurrence seen in pediatric and young adult populations, with nearly 80% occurring before the age of 20, making childhood and adolescence especially susceptible periods. The potential for long-term complications emphasizes the critical importance of accurate diagnosis, immediate therapeutic intervention, and long-term follow-up to achieve favorable prognoses. Inadequate management of these injuries can result in serious complications such as pulp necrosis, root resorption, and premature tooth loss [1,2].

Notably, Saudi Arabia has a higher reported incidence of traumatic dental injuries compared to other countries, suggesting a particular challenge in this region. In Riyadh, Saudi Arabia, significant rates of dental trauma among adolescents, specifically 34% of boys aged 12-14 years, have been reported [3].

Extrusive luxation, also termed peripheral displacement or partial avulsion, involves the partial displacement of a tooth from its socket. Radiographically, this injury is characterized by an increased width of the periodontal ligament (PDL) space. Such trauma indicates damage to the tooth’s neurovascular supply and the surrounding supporting structures [4].

Following dental injuries, root resorption emerges as a significant complication [5]. Odontogenic hard tissues are physiologically protected from resorption by the presence of precementum on the radicular surface and predentin lining the pulp chamber. Traumatic dental injuries can compromise the integrity of the tooth’s protective barriers and expose the underlying mineralized dentin and cementum to the activity of odontoclasts, which then initiates a resorptive cascade [6].

The early presentation of root resorption is commonly asymptomatic and may only be detected accidentally through routine radiographic examination. With pathological advancement, symptoms such as pain, discoloration of the tooth, or increased mobility may become evident. Therefore, timely and accurate diagnosis is critical to improve the long-term prognosis of traumatized teeth [7].

Concurrent traumatic dental injuries, such as crown fractures combined with luxation injuries, significantly increase the risk of adverse pulpal outcomes. This increased susceptibility stems from the combined impact of a crown fracture, which compromises pulpal integrity and introduces potential bacterial contamination, and the luxation injury, which disrupts or severs the neurovascular supply to the pulp. This combined effect substantially elevates the potential of pulp necrosis and subsequent infection of the root canal system. Consequently, the damaged root surface, a direct result of the luxation and any necessary repositioning or replantation, becomes more vulnerable to external inflammatory resorption due to the presence of an infected root canal system [8].

## Case presentation

**Figure 1:**
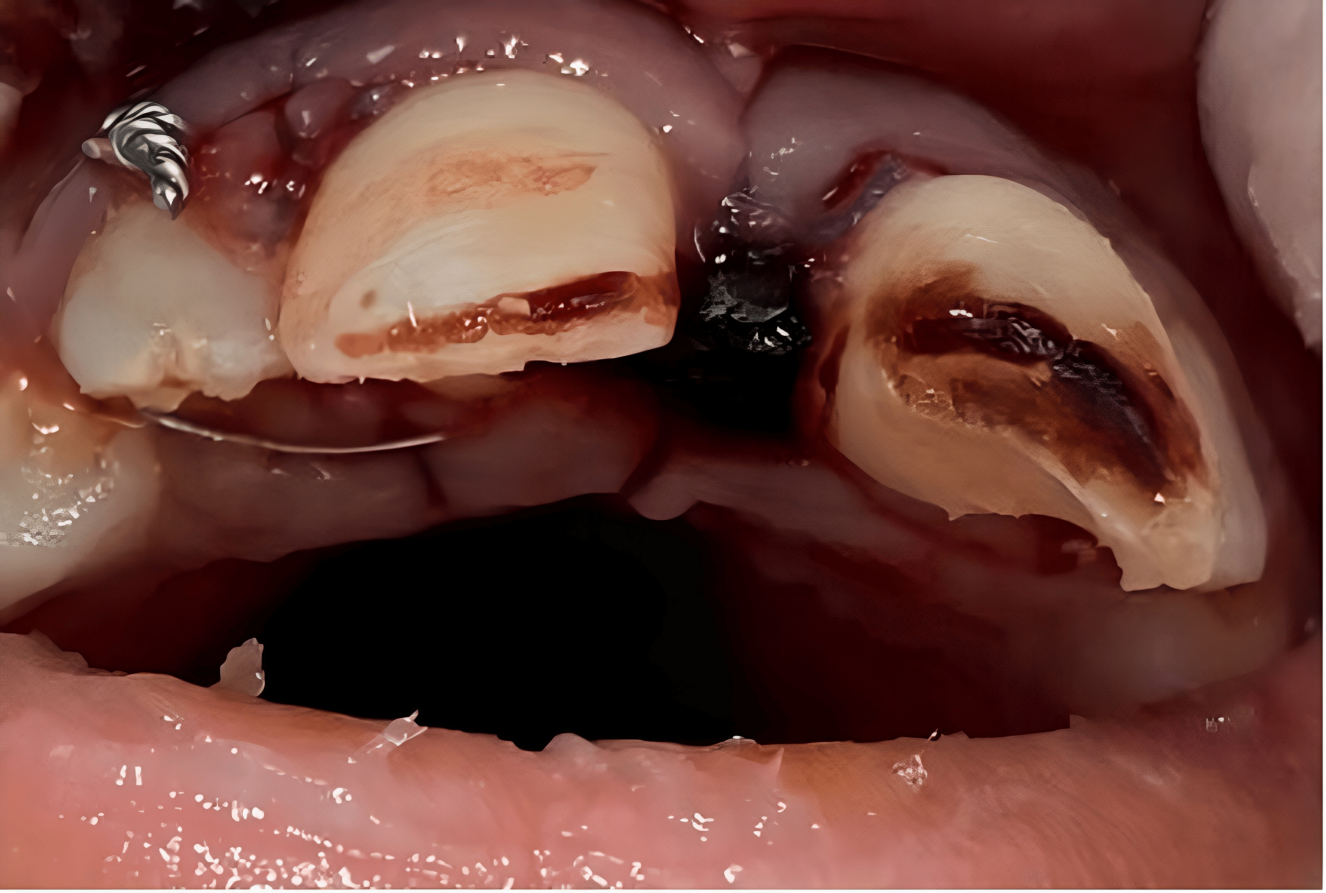
Preoperative clinical photo. Preoperative clinical photo showing teeth #12 and #11 were splinted together using a metal wire, while teeth #21 and #22 were splinted together using another metal wire.

A nine-year-old healthy boy was referred from the OMFS department in the Armed Forces Hospital, Khamis Mushait, for management of traumatized anterior permanent teeth. The patient was pushed while playing in school, and the two upper central incisors were partially pulled outside of their sockets. Upon clinical examination, teeth #11 and #21 had extrusive luxation injury and were displaced axially. Tooth #21 had an uncomplicated crown fracture. Teeth #12 and #11 were splinted together using a metal wire, while teeth #21 and #22 were splinted together using another metal wire, causing a large gap between the two upper central incisors (Figure 1).

**Figure 2:**
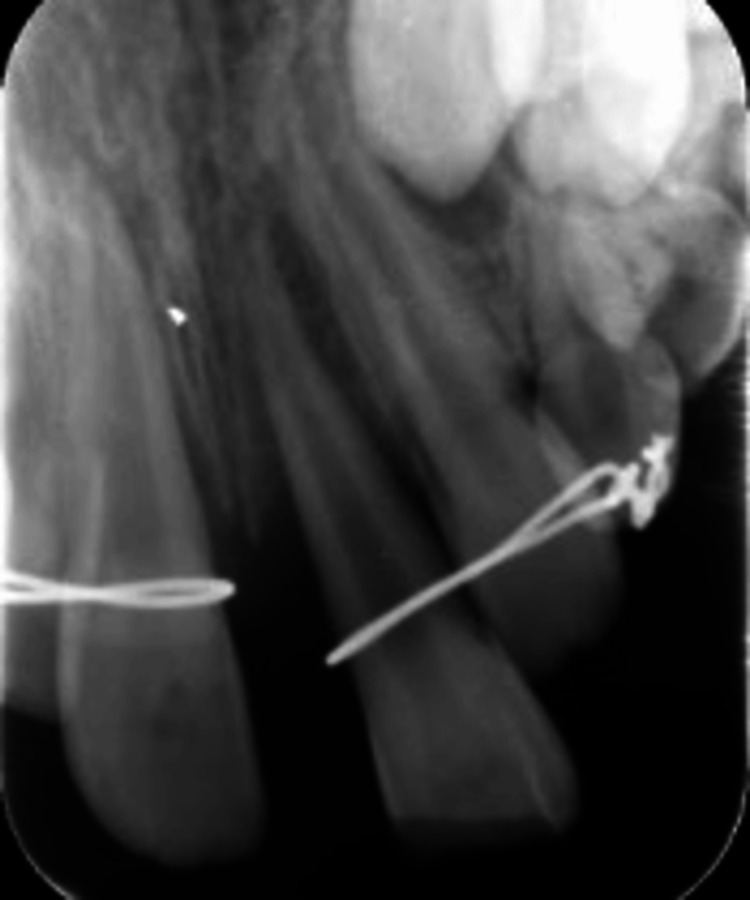
Preoperative periapical radiograph. Preoperative periapical radiograph after trauma showing teeth #12 and #11 were splinted together using a metal wire, while teeth #21 and #22 were splinted together using another metal wire. The radiograph also shows teeth #11 and #21 having open apices.

**Figure 3:**
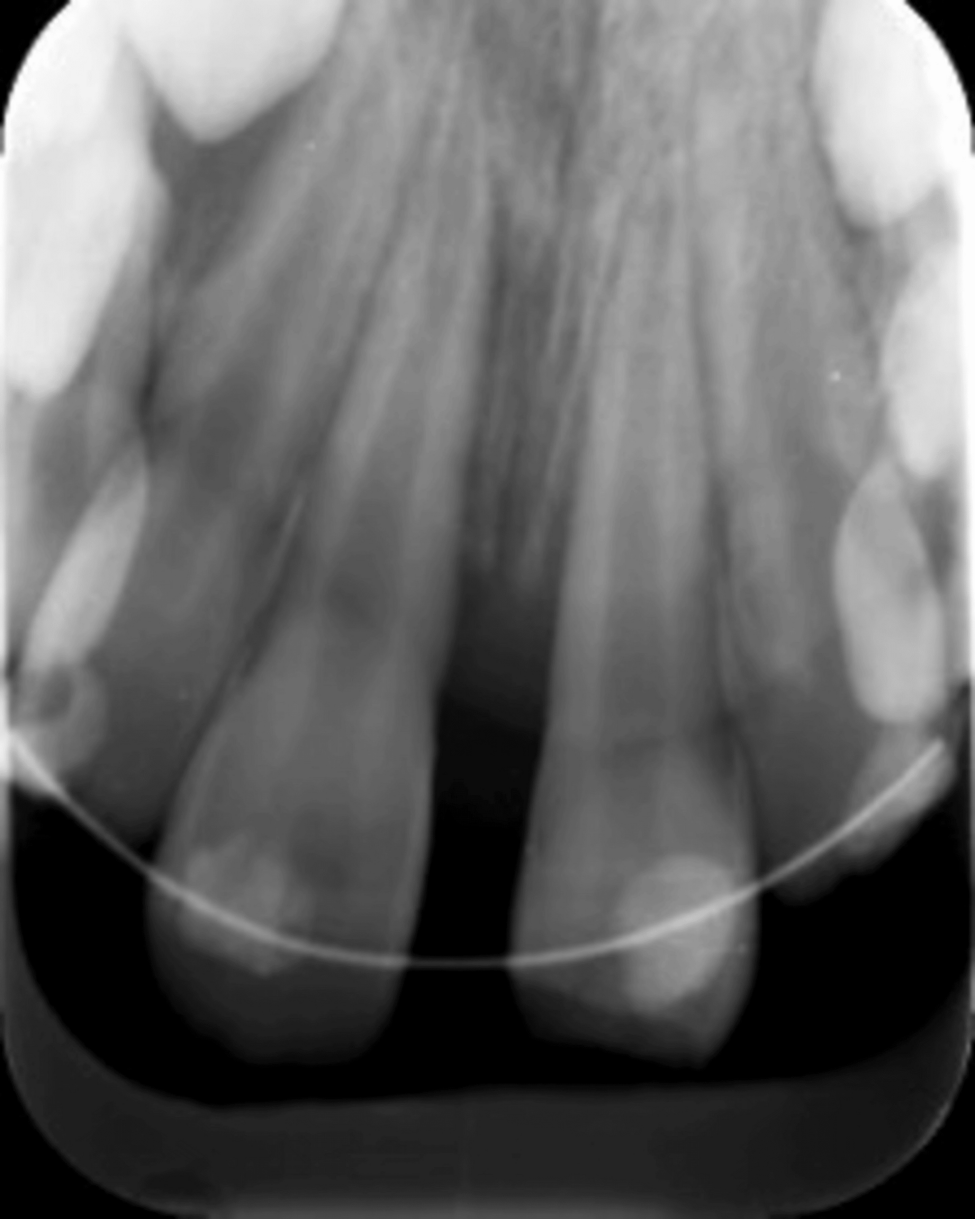
Periapical radiograph after splinting. A periapical radiograph showing teeth #12, #11, #21, and #22 after repositioning and splinting using a flexible orthodontic wire.

A grade II mobility was evident on both #11 and #21, along with pain on percussion and palpation. Tooth #21 did not respond to the sensibility test, while teeth #12, #11, and #22 responded normally. Teeth had open apices, as shown in the preoperative periapical radiographs (Figure 2). Under local anesthesia, repositioning and splinting with a flexible orthodontic wire were done (Figure 3).

**Figure 4:**
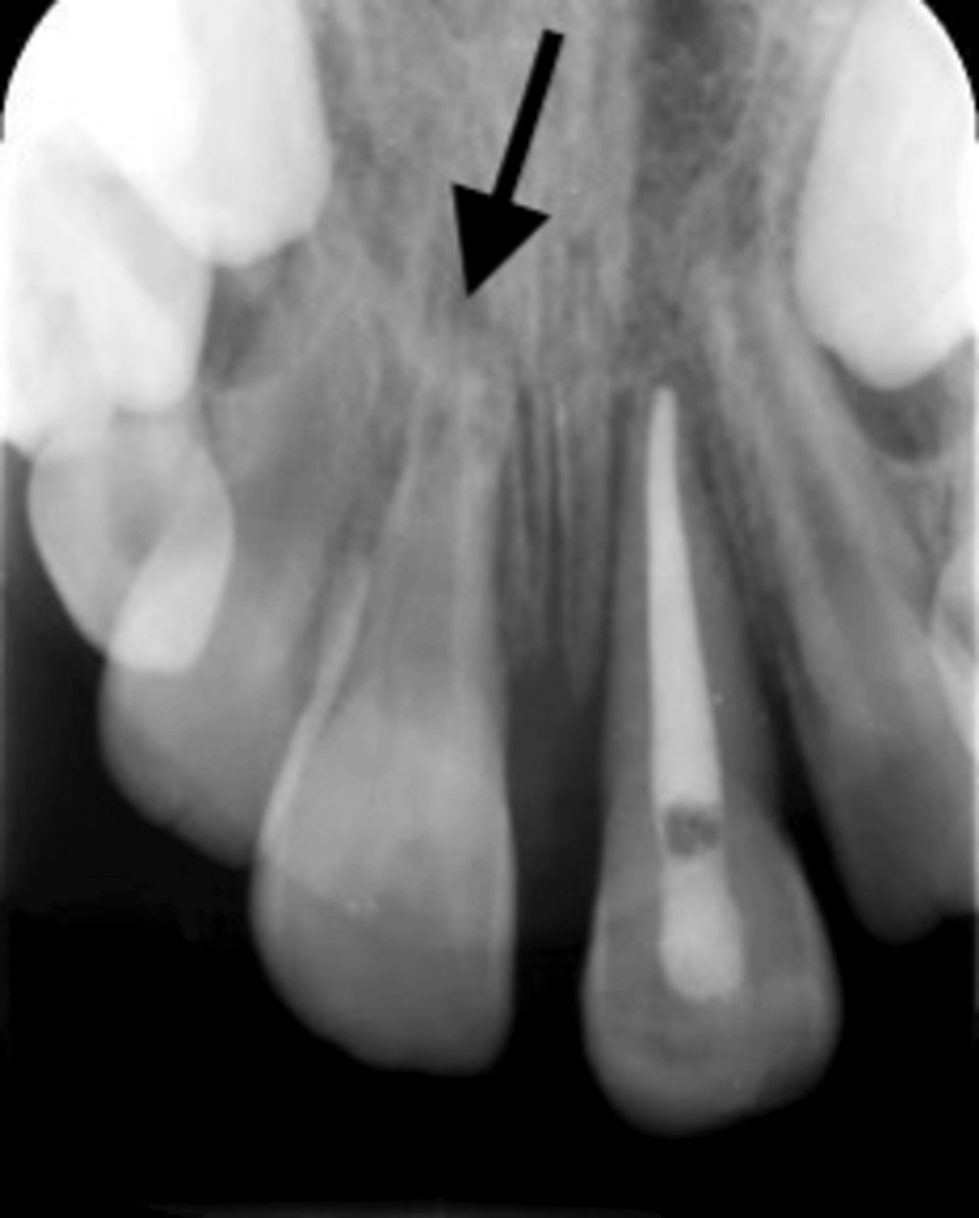
Periapical radiograph showing signs of root resorption for teeth #11 and #21 after root canal treatment. A periapical radiograph showing signs of traumat-induced external inflammatory root resorption related to teeth #11 and #21 after receiving root canal treatment.

Treatment options were discussed with the patient’s guardian, and root canal treatment of teeth #11 and #21 was suggested to prevent trauma-induced inflammatory root resorption. However, the patient’s guardian opted for root canal treatment for tooth #21 only and decided to keep tooth #11 under observation as it was vital and responded normally to cold tests. Follow-up in two, four, six, and eight weeks was planned. Tooth #21 received root canal treatment using a bioceramic apical plug, and the canal was backfilled with thermoplasticized gutta-percha. On the eighth-week follow-up visit, signs of root resorption for tooth #11 were evident, and root canal treatment initiation was planned the next day (Figure 4).

**Figure 5:**
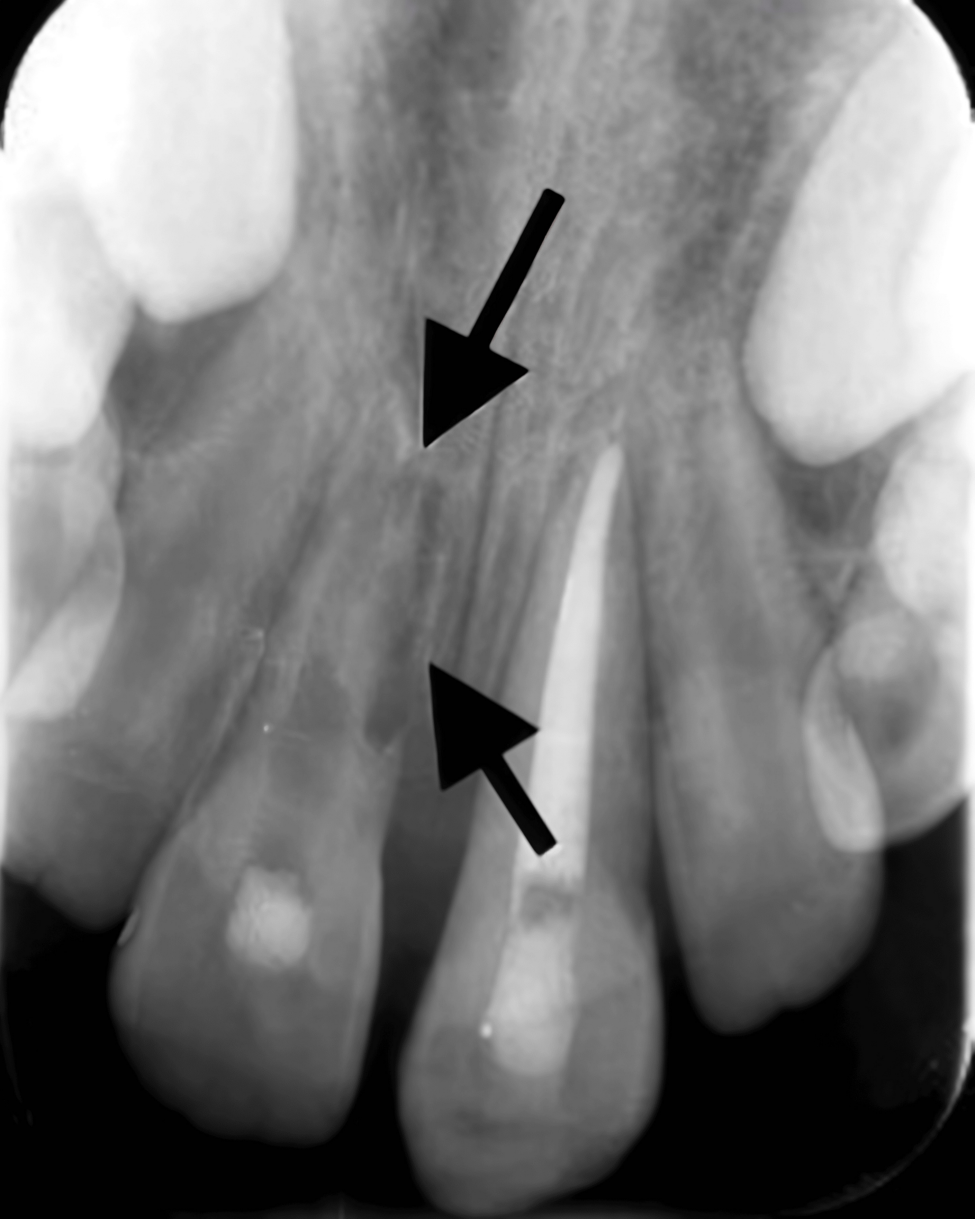
Periapical radiograph showing aggressive root resorption on tooth #11. A periapical radiograph showing progression of trauma-induced external inflammatory root resorption related to tooth #11.

**Figure 6:**
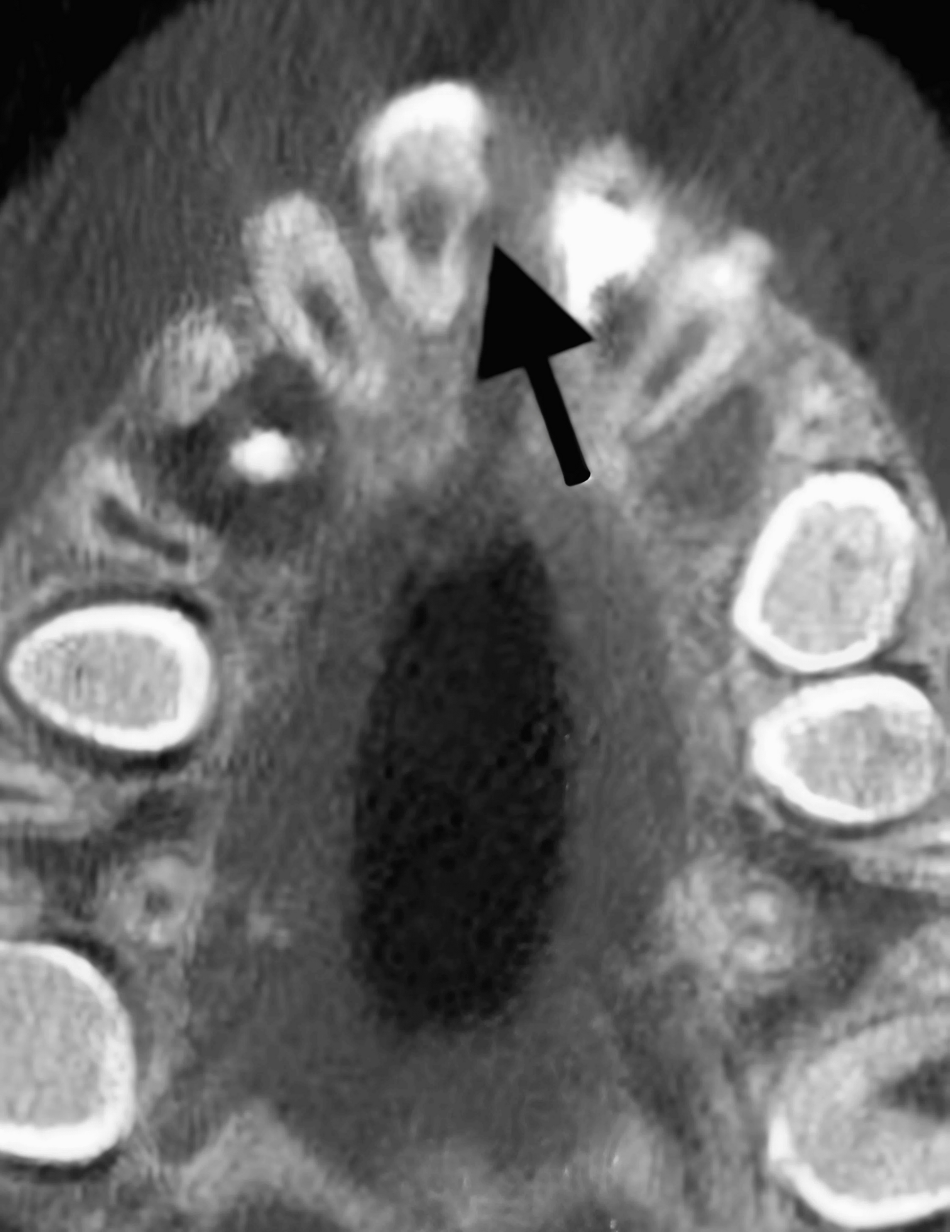
Cone-beam CT showing aggressive root resorption on tooth #11. A cone-beam CT radiograph showing progression of trauma-induced external inflammatory root resorption related to tooth #11.

**Figure 7:**
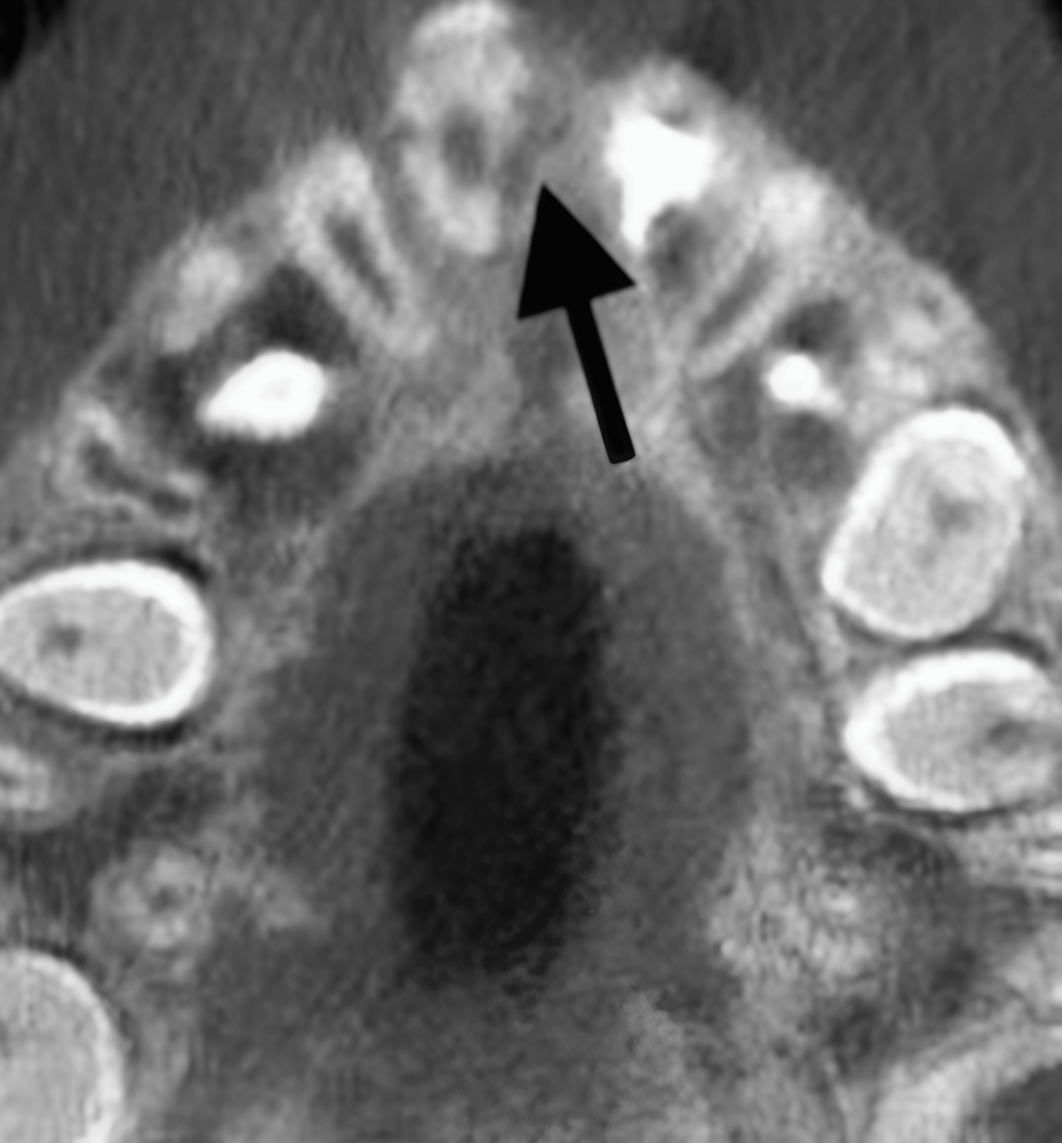
Cone-beam CT showing aggressive root resorption on tooth #11. A cone-beam CT radiograph showing progression of trauma-induced external inflammatory root resorption related to tooth #11.

**Figure 8:**
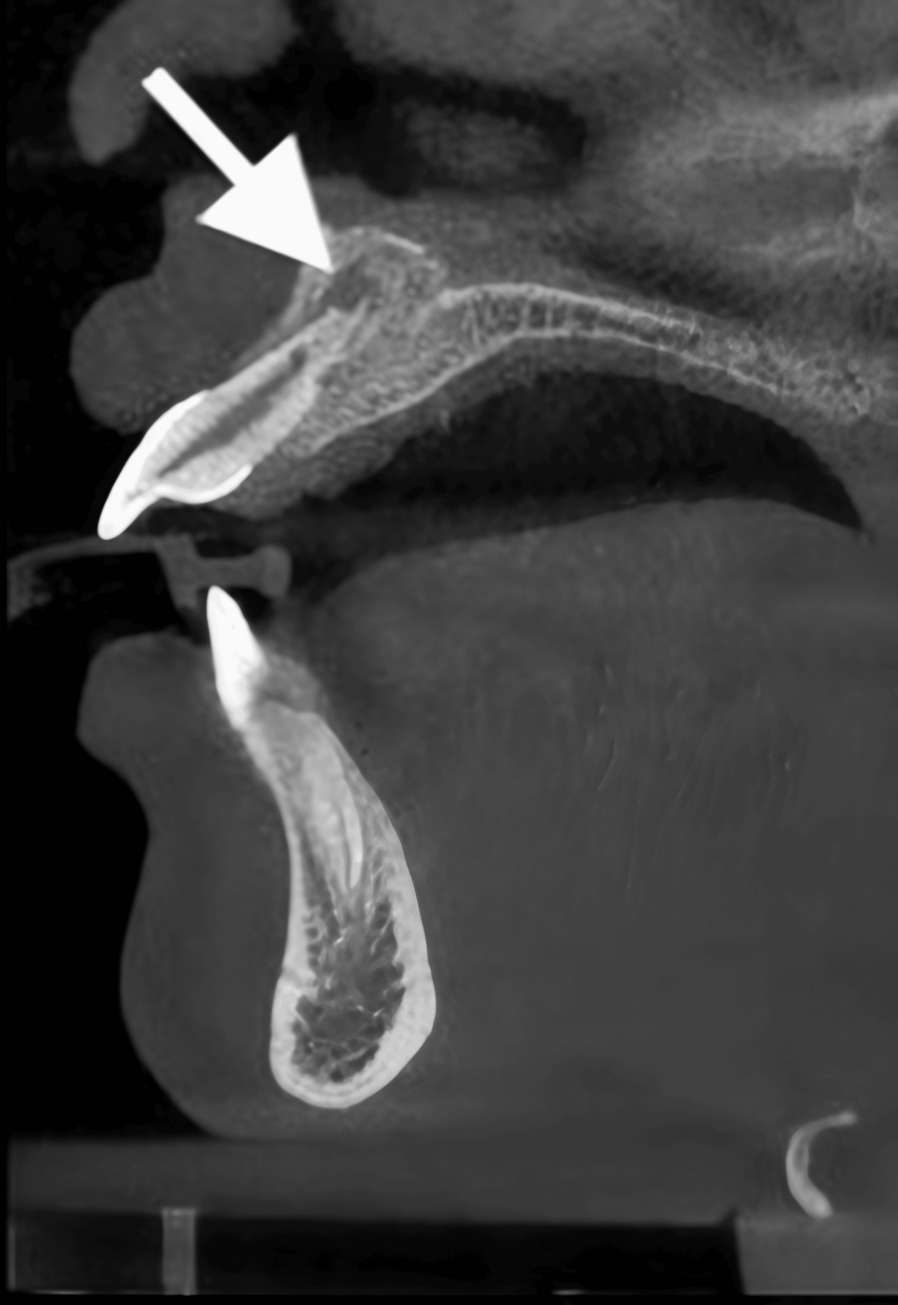
Cone-beam CT showing aggressive root resorption on tooth #11. A cone-beam CT radiograph showing progression of trauma-induced external inflammatory root resorption related to tooth #11.

**Figure 9:**
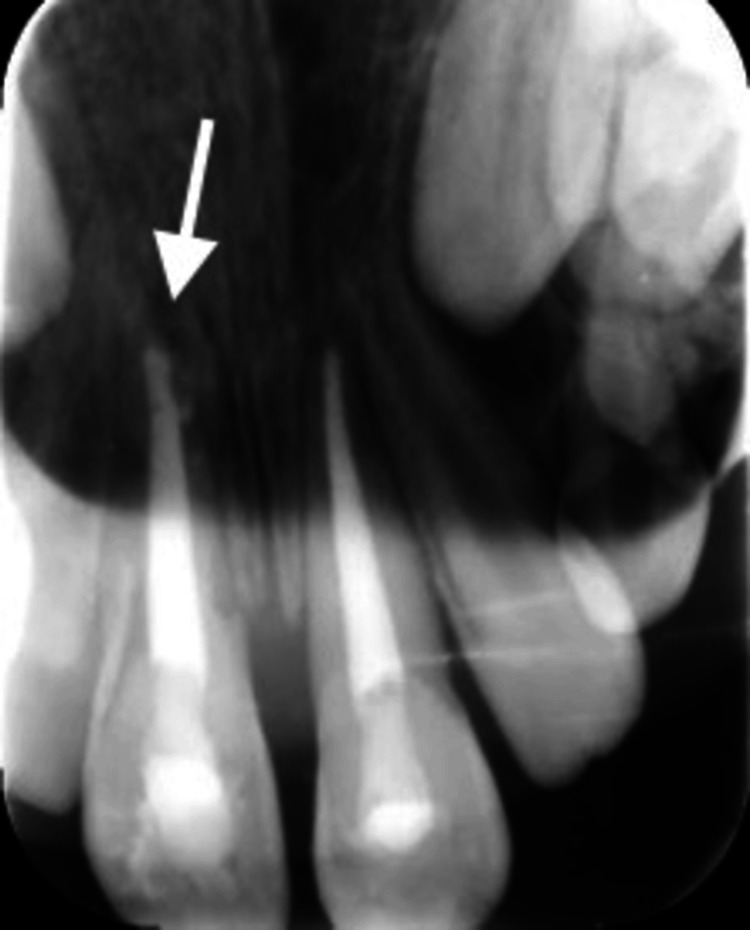
Periapical radiograph after root canal treatment of tooth #11. A periapical radiograph showing tooth #11 after root canal treatment using bioceramic putty.

However, the patient could not come back to the clinic until three weeks later, when resorption quickly progressed. Calcium hydroxide intracanal (DENTSPLY, AH temp calcium hydroxide-based root canal dressing) dressing was used every two weeks until resorption stopped, and the root canal space was filled with bioceramic putty (FKG, Total Fill BC RRM Fast Set Putty premixed-ready to use) (Figures 5-9).

**Figure 10:**
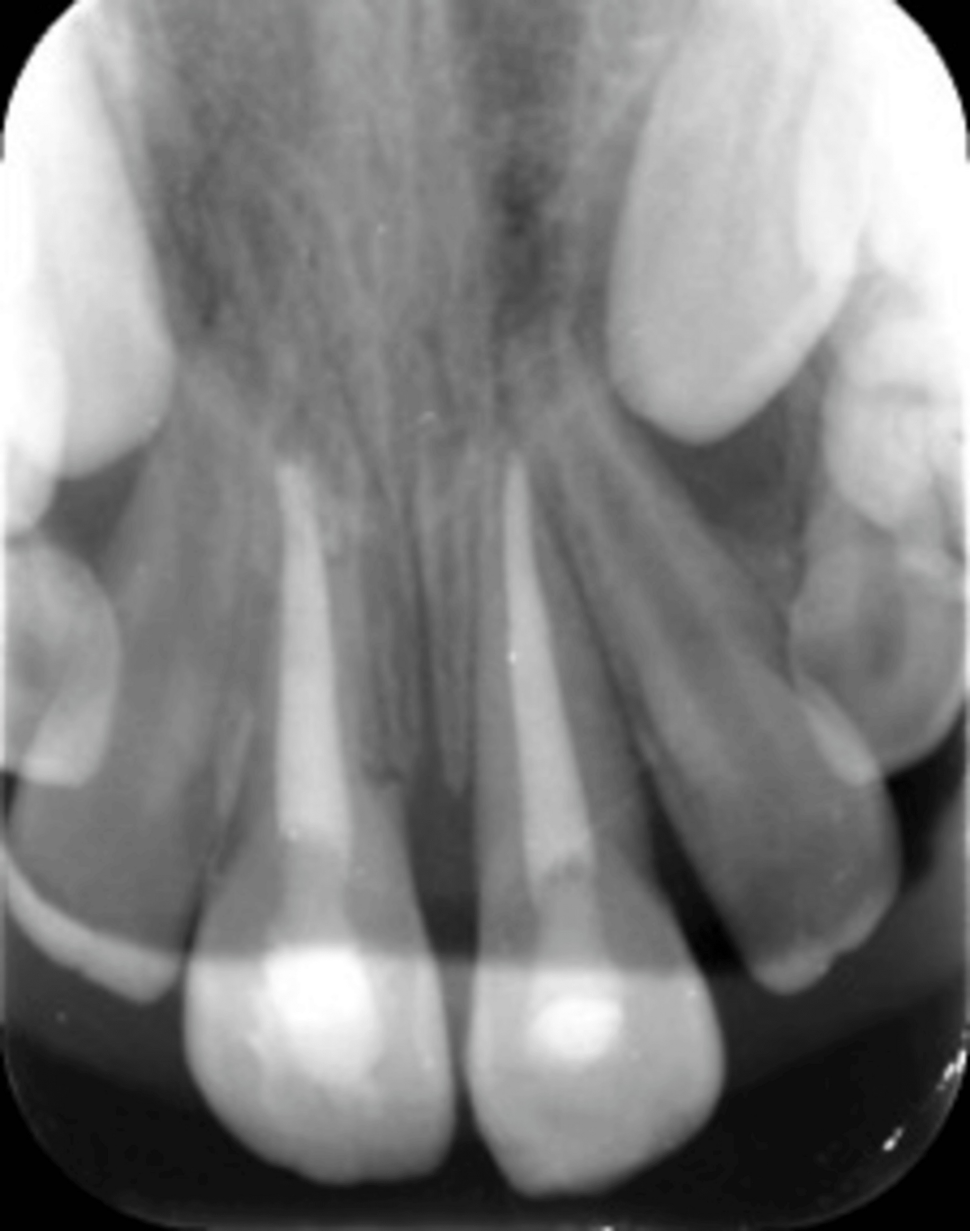
Periapical radiograph #11 and #21 after a three-month follow-up. A periapical radiograph showing teeth #11 and #21 after a three-month follow-up.

Teeth #11 and #21 were sealed with esthetic composite resin restoration. A three-month follow-up demonstrated stability of the resorption site, along with periapical healing in the absence of clinical symptoms and mobility (Figure 10).

## Discussion

Following dental trauma, various types of root resorption can emerge. External inflammatory root resorption (EIR) is recognized as one of the most destructive variants, frequently following profound luxation or avulsion injuries. Its destructive cascade initiates with PDL compromise and is complicated by bacterial infiltration from a necrotic dental pulp. Toxins from these bacteria permeate dentinal tubules, triggering an inflammatory reaction within the PDL that, in turn, activates odontoclasts, leading to relentless root breakdown. EIR typically appears on radiographs as a distinctive “bowl-shaped” radiolucency spanning both the affected root and adjacent bone. The urgency of EIR diagnosis cannot be overstated due to its swift advancement, necessitating the use of advanced imaging, such as cone-beam CT, for accurate diagnosis, as standard two-dimensional views are prone to underestimating the lesion’s actual size [9].

Optimal management of extrusive luxation is paramount to improve prognosis and avert complications such as pulp necrosis, root resorption, and the loss of supporting bone around the tooth. Even though immediate action is ideal, unfortunately, it is not unusual for treatment to be delayed. This often stems from parents lacking awareness, challenges in diagnosis, or limited access to specialized dental services [10]. The displaced tooth should be immediately repositioned with finger pressure and then stabilized with a passive, flexible splint for two weeks to promote PDL repair [1].

In immature teeth with open apices, the pulp may survive and heal, and spontaneous pulp revascularization may occur. Therefore, the recommendation is to avoid root canal treatment unless there is clinical or radiographic evidence of pulp necrosis or periapical infection on follow-up examinations. However, the risk of EIR should always be weighed against the chances of obtaining pulp space revascularization. Regular follow-ups are mandatory to ensure that root canal treatment can be commenced as soon as this type of resorption is detected [1].

In mature teeth with closed apices, pulp necrosis is a common complication. Calcium hydroxide is recommended as an intracanal medicament for up to four weeks, followed by root canal filling. Alternatively, a corticosteroid/antibiotic paste can be used as an anti-inflammatory and anti-resorptive intracanal medicament to prevent EIR. This paste should be placed following repositioning and left in situ for at least six weeks [1].

The role of calcium hydroxide in modifying root dentin pH has been studied, particularly concerning its application in external inflammatory resorption. When used as a root canal medicament, calcium hydroxide facilitates the diffusion of hydroxyl ions through dentinal tubules and cementum into the PDL. This diffusion is enhanced following traumatic cementum removal or surface resorption, leading to a greater concentration of hydroxyl ions reaching the PDL and bone. Notably, the resultant pH in the outer dentin can attain levels between 8.0 and 9.5, a concentration detrimental to the attachment and proliferation of PDL fibroblasts, which are inhibited above a pH of 7.8 [8].

In incompletely developed teeth, avulsion with crown fracture and intrusion with crown fracture are the injuries that present a high chance of pulp necrosis and root resorption. While in fully developed teeth, avulsion, intrusion, lateral luxation with crown fracture, and extrusion with crown fracture are the injuries that are most likely to result in infection of the root canal system and root resorption, according to the stage of root development at the time of injury. The decision to immediately initiate root canal treatment in fully developed roots to prevent root canal treatment is considered a preventive measure, while keeping these teeth under observation and delaying the initiation of root canal treatment until signs of EIR are evident is considered an interceptive management [8].

Immediate root canal treatment following repositioning/replantation and splinting should be considered for teeth with these injuries to prevent the development of EIR [8]. In this case report, tooth #21 received root canal treatment to prevent EIR, while tooth #11 received interceptive treatment to stop the progression of EIR [8].

## Conclusions

This case report underscores the profound impact of timely and appropriate intervention in the management of trauma-induced root resorption following dental injuries. It serves as a compelling reminder for clinicians regarding the unpredictable and often rapid progression of root resorption post-trauma. It reinforces the paramount importance of immediate and comprehensive diagnostic evaluations, proactive preventive strategies, and vigilant follow-up. Any delay in addressing predisposing factors or established resorption can significantly compromise the prognosis and potentially lead to premature tooth loss. Therefore, a high index of suspicion and an unwavering commitment to prompt intervention are indispensable in mitigating the long-term complications of traumatic dental injuries.
